# Born to donate: proposals for “savior sibling” regulation in Latin America

**DOI:** 10.25100/cm.v49i2.3619

**Published:** 2018-09-30

**Authors:** Alejandra Zúñiga-Fajuri

**Affiliations:** Centro de Investigaciones de Filosofía del Derecho y Derecho Penal, Escuela de Derecho, Universidad de Valparaíso. Valparaíso, Chile. Universidad de Valparaíso Centro de Investigaciones de Filosofía del Derecho y Derecho Penal Escuela de Derecho Universidad de Valparaíso Valparaíso Chile

**Keywords:** Non-therapeutic medical interventions, Children organ transplant, bioethical legislation

## Abstract

A Savior Sibling is a child who is born to provide an organ, bone marrow or cell transplant, to a sibling that is affected with a fatal disease. There are created with the *in vitro* fertilization and pre-implantation genetic diagnosis and, in the process, the ethical standards for organ donation of children become less demanding. Therefore, we propose that the authorization of the technique considers, unavoidably, the opinion of an impartial third party that can guarantee the welfare of the child. We develop a critical analysis of the laws that regulate the creation of babies to serve as organ donors. We evaluate under what circumstances the organizations that play a part in the decisions, fulfill the ethical standards to allow the organ donation of children.

## Introduction

It is common that advances in medicine and bio-medical research occur faster than bioethical ruminations concerning their morality. This may provoke a sense of *fait accompli* concerning these advances, wherein the concerns of philosophers, politicians and legal theorists are easily written off as useless. We rarely question the assumption that “scientific breakthroughs” are inherently positive, and the case of savior siblings is no exception.

The term “savior sibling” generally describes a baby that has been created through In Vitro Fertilization (IVF), and in accordance with a Pre-Implantation Genetic Diagnosis (PGD), in order to act as a donor for a sick sibling. Since hematopoietic stem cells are found in the bone marrow, peripheral blood and umbilical cord blood, the sibling will be used, from the moment of his/her birth, in order to donate umbilical cord blood or for more invasive procedures, such as multiple bone marrow transplants or, even, organ transplants ^1^.

The Pre-Implantation Genetic Diagnosis to create donor children is a protocol that has yet to be developed in Latin America. For this reason, the main objective of this paper is to analyze the PGD process and consider how it could pose a danger to the universally agreed-upon ethical norms for organ donation by those incapable of giving consent. 

Although the creation of savior siblings has been the object of considerable bioethical debate, an important number of experts and specialists have concurred that it is an ethically admissible practice ^2^^-^^7^. Additionally, it is a technology that has been legally incorporated into different legislation in North America, Europe and Australia ^5^^,^^8^. For this reason - and although the bioethical discussion on the moral legitimacy of this procedure can and should remain open - in this paper, I will concentrate on the basic rules that countries should define, in the case that they should decide to authorize and regulate the creation of savior siblings*.* At the same time, I will also attend to concerns regarding what have been called *viability restrictions,* such as those theories that promote unreal moral demands that ignore human psychology and/or the monetary and institutional incentives to which an individual responds ^9^.

Since this is a “scientific breakthrough” that is here to stay and has been staunchly defended on many fronts - legal, bioethical, and medical - it does not seem reasonable to insist on outlawing it. Concurrently, the following arguments against the creation of savior siblings will not be analyzed, apart from, perhaps, a few indirect references:

1. That PGD and IVF are ethically questionable procedures because they imply the *destruction of human embryos* ^10^ *.*
2. That children cannot be treated as objects since this violates the categorical *Kantian moral principle* which states that “as an end in itself humans are required never to treat others merely as a means to an end, but always, additionally, as ends in themselves” ^11^.
3. The argument that allowing the techniques that produce savior siblings will lead to the creation of a *slippery slope* that will end up promoting genetic selection in the search for “perfect babies” ^12^ ^,^ ^13^.

The objective of this paper, then, is to argue that it is necessary an explicit authorization for the procedures that lead to the creation of savior sibling, which involve the intervention of an impartial third party capable of protecting the well-being of the future child. In order to do this, we will divide this paper into three sections. First, a section that seeks to explain what the procedure is for the creation of savior sibling, its history and the bioethical problems related to it that have been identified in the bibliography and medical practice. In the second section, I will revise the regulations in place in those countries that, in one way or another, have authorized the procedures that lead to the creation of savior sibling -PGD and IVF. Here I describe the requirements that legislation, the courts and ethics committees have demanded when seeking to regulate this practice and how these requirements have changed over time. Finally, in the third section, I analyze the opposing positions to my argument, and conclude stressing the importance of always considering an impartial third party opinion when deciding whether to donate organs and tissues from a child or one who is legally incompetent. 

## 1. Creating savior siblings 

David Benatar defines non-therapeutic pediatric interventions -such as the extraction of a baby’s bone marrow - as medical interventions that do not have as their goal preventing or curing illness. They do not seek to benefit the child who is being intervened but, rather, someone else ^14^. The moral problem of non-therapeutic pediatric interventions lies in the irrefutable fact that they lie outside of the generally accepted borders of the minimum conditions needed for medical interventions in people who cannot consent. Children, obviously, cannot manifest valid consent at an early age, so their legal representatives must do so when considering treatments that will directly benefit them. These could be seen as cases of justified paternalism. However, medical interventions that *do not provide a direct benefit* to the child are more difficult to justify and this occurred in the case that I will present here.

In 1996 Lisa and Jack Nash’s 4-year-old daughter, Molly, was diagnosed with a severe genetic disease, known as Fanconi Anemia (FA). This illness affects the production of red blood cells, leukocytes, and platelets, promoting hemorrhage, as well as bone marrow abnormalities. The only known cure for FA today is the transplant of hematopoietic stem cells (HSC), for which there must be a donor with a perfect genetic match, capable of replacing the affected immune system.

Following their doctor’s advice, the Nash family decided to go forward with the innovative procedure of Pre-implantation Genetic Diagnosis (PGD), which would allow them to conceive a baby free from the hereditary pathology that affected Molly and genetically compatible with her, in order to be able to use the baby’s umbilical cord blood for his sister’s transfusion ^15^. As a result of this process, on August 29, 2000, the first *Savior Sibling* in the world was born in the U.S., named Adam, and whose successful transplant would save his sister’s life ^16^.

In the U.K., there was also the case of the Hashmi family, where they requested authorization for the creation of a *Savior Sibling* that would allow them to cure the illness of the child Zain Hashmi, who had a severe genetic disorder in his blood that required regular blood transfusions and large quantities of medication. It was hoped that all of this could be solved with the transfusion of a *Savior Sibling’s* umbilical cord stem cells.

Similar to the Nash family case, the Hashmi family was also motivated by two factors: conceiving a child free from a genetic disease and making sure that that new baby could be a suitable donor for his/her brother. The responsible U.K. health authorities (The Human Fertilization and Embryology Authority, HFEA) approved the procedure, underscoring how it complied with the basic requirement for allowing Pre-Implantation Genetic Diagnosis: that of being “therapeutic”, as they would be selecting an embryo free from an inherited genetic disease. However, in another case, and following these same criteria, the HFEA denied authorization to the Whitaker family, since their sick son (Charlie) did not have a hereditary disease whose prevention could justify the Pre-Implantation Genetic Diagnosis ^17^.

## 2. Regulating savior siblings

After the Nash family’s successful experience, demand for PGD, in order to transplant stem cells from children to sick siblings, has skyrocketed and is likely to continue growing. The most frequent medical interventions used have been blood transfusions, bone marrow transplants and organ transplants. These last two types of intervention clearly bring with them certain risks and harm to the baby, without producing a direct benefit for him or her. 

Of the 15 countries in Western Europe, 13 allow PGD. Some, including the U.K. and France, have allowed this for a long period of time around 20 years ^18^. The countries that have only recently permitted it are Germany - where it is legal since 2011- and Switzerland, which has allowed it since September 2017 (when it emerged victorious in a referendum) ^19^. In Latin America, there is Brazil and its Decree 1321, from December 2015 -*National Transplant System’s Technical Regulations* - which states that “the use of hematopoietic stem cells (bone marrow, peripheral blood, or umbilical cord blood) should consider the risks for the donor and the risks and the benefits for the recipient” ^20^.

In general, then, regulation of this procedure centers on two related aspects concerning the future baby’s well-being. First, that the PGD has a therapeutic objective and, second, that the substitution of the sibling’s consent complies with “best interest” criteria, common to cases of consent substitution with those who are legally incompetent. For example, one must guarantee that the benefit to the child is greater than the risk of the intervention and that this intervention does not create excessive harm.

### 2.1 PGD’s therapeutic objective 

Regulations in the U.K., Spain, and Australia make explicit the demand of complying with the two requirements recently mentioned for authorizing the creation of a savior sibling: having a therapeutic objective and complying with the “best interest” principle. In the U.S. where there is not federal regulation concerning this matter, authorization has centered only on the second requirement, that of “best interest”, which will be reviewed in the following section ^21^.

In the case of Australia, even when regulation may vary between states, the general rule is to permit PGD only when, in addition to producing genetic compatibility with the sick sibling, it also prevents, “a real risk to the future embryo that it suffer a severe genetic disease” ^17^. This is similar to the regulation that existed in the U.K. before a modification that the “Human Fertilization and Embryology Authority” (HFEA) made in 2005, which states that, “The law also permits tissue typing if the embryo will not, in addition to the histocompatibility test, be tested for a particular genetic or mitochondrial abnormality” ^22^.

Spanish legislation regulates the use of the procedure through its *Assisted Reproduction Procedures Law (Ley de Técnicas de Reproducción Humana Asistida),* which, in Article 12.2, defines in which situations embryos can be examined in order to test histocompatibility with a third party. At the same time, the Oviedo Convention establishes that, “Only in exceptional circumstances and in protective conditions covered by the law, will the extraction of renewable tissues from a person who is not able to express consent be authorized and only if in compliance with the following conditions:

- If there is no compatible donor capable of giving consent;
- If the recipient is a brother or sister of the donor;
- If the donation is in order to save the life of the recipient;
- If a specific written authorization has been given by the representatives of the case and the ethics committee” ^23^.

The contrast between regulations in the U.K. and Australia versus regulations in Spain illustrates well the question of whether or not the procedure should have a therapeutic end. While it is clear that allowing for the development of an embryo without a genetic disease will not harm a future child, the reasoning is actually the inverse: that the child born from this procedure is inherently benefitted solely due to the simple fact that without it he or she would not exist. Even though there have been cases wherein people have demanded compensation due to their births in determined circumstances (cases known as *Wrongful Birth* and *Wrongful Life*) ^24^ ^)^ the procedure should be authorized when it is shown that a healthy embryo will be selected and that he or she will be incorporated into the family and treated as another family member.

### 2.2 Surrogate consent and the “best interest” principle 

Informed consent rules for non-therapeutic interventions in adults tend to be very strict. Consent is a necessary ethical tool that should accompany all medical interventions and a therapeutic end is, generally, the objective in medicine. For this reason, the majority of medical procedures, excepting only a few cases of emergency care, require free and informed consent on the part of the patient ^14^. In the case of SS, however, we confront a non-therapeutic intervention that is carried out without the express consent of the child (instead, as we shall see, this consent is substitutive). Due to this complex situation, one of the basic requirements that must be met in order to comply with authorizations for this procedure, is that the “best interest” of the minor child will be guaranteed. As we have already mentioned, the authorization for this procedure can cover a wide range of possibilities, from umbilical cord extraction, to bone marrow transplant and, even, eventually, organ transplant ^18^.

U.S. courts have generally employed two main criteria when resolving the legitimacy and the legality of medical procedures in children and people who are legally incompetent. The *Substituted Judgment Standard,* which originated in British courts, is applied in those cases in which the person was, once, competent, so that the representative will decide in accordance with what, in his or her opinion, the patient would have wanted in that situation. On the other hand, when we are talking about individuals who have never been competent (as in the case of small children) we use the *Best Interest Standard,* derived from the authority that the law recognizes in parents over children. Parental consent in the medical context implies that, once born, the parents are legally able to consent to a non-therapeutic medical intervention in the name of the newborn ^18^.

In the U.K. it has been decided that the child’s “best interests” are not circumscribed solely to the medical, but, rather, that they can include the interests of the entire family and those of his/her siblings; that is, that the analytical criteria should be wider in scope ^17^ ^,^ ^25^. In accordance, when deciding about compliance with the “best interest” principle in cases of organ donation by those incapable of giving consent, the courts also weigh the possible psychological and physiological effects that result from the intervention with those that could be produced due to the death of a sibling ^13^ ^,^ ^21^.

 The “best interest” principle cannot erase, obviously, the risks associated with a medical procedure. Both bone marrow and live organ transplants have been related to a number of psychosocial and physiological risks for both donors and recipients. HSCT studies on infant donors show an increase in stress and anxiety and lower self-esteem in donor siblings, as well as moderate levels of post-traumatic stress ^17^ ^,^ ^25^. The physiological problems that infant donors face often have to do with the medicines used for anesthesia during the transplant procedure and the adverse effects of the transplant itself. In the case of bone marrow transplant, effects such as fatigue, pain at the site of extraction, lumbago, headaches, nausea, difficulty walking, sleep problems, and, less commonly, bleeding, have all been observed ^20^ ^,^ ^26^.

In the case of organ donors, in addition to the evident disadvantage of living with one less organ, there have also been reported risks of infection, temporary or permanent disability and, even, death ^21^ ^,^ ^27^. In conjunction with this, there is also the risk of psychological suffering, of feeling resentment or depression as a result of the donation or due to transplant failure. On the other hand, there are also those that have a positive psychological reaction, showing signs of closeness with the recipient and a sense of having contributed to the family ^13^ ^,^ ^21^.

Associations of healthcare professionals, such as the U.S. Live Organ Donor Consensus Group and the American Academy of Pediatrics (AAP), have also considered the legality and the legitimacy of organ donation on the part of minors. Although organ donation by children is rejected, on principle, they have admitted exceptions if four requirements are met:

1. when there is evidence that the potential donor and recipient will be benefitted;
2. when the surgical risks for the donor are extremely low;
3. when all other resources have already been exhausted (for example, it is not possible to use organs donated by an adult); and
4. when there does not exist the time nor the effective possibility of receiving through a deceased donor. Sometimes a fifth requirement is also considered: that the psychological and emotional risks for the donor child are minimized ^13^ ^,^ ^21^.

## 3. Organ donation by the legally incompetent: is it really that much of an exception?

In the U.S., children have been a possible source of organ donation for quite some time. Starting in 1954, with the first successful kidney transplant between identical twins, and followed by another three transplants in 1956, the United Network for Organ Sharing (UNOS) has revealed that at least 60 children under 18 years old have been live kidney donors between 1987 and 2000 and another four have been living liver donors since 1989. In the hotly debated *Little v. Little* Case (576 S.W.2d 493) the Texas State Court of Appeals allowed for the authorization of a kidney transplant from a 14-year-old girl with Down’s Syndrome to her sick brother, specifically invoking the “parental authority” of the parents and the general idea of there being an indirect benefit to the donor ^28^.

Today there continues to be a considerable number of cases of underage organ donation between siblings in the U.S. In fact, in California, up until 2006, 12% of all organ donors were under 18 years old ^29^. In this, the U.S. is not unique to North America. It also exists in Canada, were the first case occurred in 1958, when a 15-year-old girl donated a kidney to her twin sister ^30^.

The World Health Organization has issued the following edict that, “No cells, tissues or organs should be removed from the body of a living minor for the purpose of transplantation other than in narrow exceptions allowed under national law. Specific measures should be in place to protect the minor and, wherever possible the minor’s assent should be obtained before donation. What is applicable to minors also applies to any legally incompetent person” ^31^. In the E.U., the most important legal documents that discuss organ and tissue donation are the Directives 2004/23/ CE and 2010/53/ UE. Since the E.U.’s principle goal with these is establishing a regulatory framework for ensuring the quality and safety of organs, tissues and cells, the rules concerning donation by minors are very general, leaving to member states the passage of more specific regulation. In this manner, organ donation on the part of minors is permitted in Belgium, Ireland, Luxembourg, Norway, Sweden, and the U.K ^32^.

The 1995 Transplant Law in Sweden regulates live tissue and organ donation on the part of minors. Donation is permitted if the child is related to the proposed recipient and if a suitable, compatible adult donor has not been found ^33^. In England, Wales and North Ireland, there is no age limit for when people can be considered live donors ^34^ and, in Norway, they only demand that live organ donation be restricted to those minors who are able to consent ^33^. Outside of Europe, there have been cases of kidney and liver donations on the part of children in countries such as Japan and South Korea ^35^ ^,^ ^36^. 

In Latin America there have also been cases of donors incapable of giving consent who have been authorized to be organ donors, for example in Brazil ^37^. In Argentina, there exists jurisprudence that dates from 1980 (the Saguir and DIB Case, 6-980), in which a kidney transplant was authorized from a legally incompetent person to his/her sick sibling. More recently, in a report made by the National Assistance Program for People with Disabilities in their Relations with the Justice System (*Programa Nacional de Asistencia para las Personas con Discapacidad en sus Relaciones con la Administración de Justicia*, ADAJUS), a person with Down’s Syndrome (Jorge Gandur) was declared competent to give consent for donating an organ (a kidney) to his brother, Alfredo ^38^.

Although there do not exist documented cases on the incorporation of PGD in the production of savior sibling in Latin America, consulting and comparing the international rules on organ transplant will, no doubt, prove extremely relevant to the conversation when seeking to regulate the inclusion of this procedure.

## 4. Some final reflections 

At the beginning of this paper, I stated that the Pre-Implantation Genetic Diagnosis to create donor children is a procedure that, as far as I know, has not been developed yet in Latin America. For this reason, the objective of the research presented here has been to analyze the procedures used for creating a SS, its regulation, and the ethical norms that have been agreed upon with regard to the donation of organs on the part of those who cannot consent, such as minor children. With this in mind, we have reviewed some of the laws that authorize this procedure and that allow us to make some conclusions concerning the basic principles and rules that should be adopted in Latin American countries. 

 i) Firstly, future regulation should first make decisions about IVF and PGD, and, then, proceed to regulating the donation of organs on the part of those who are legally incompetent. With regard to the first point, we must first decide whether or not IVF and PGD will only be used in order to create a savior sibling and, additionally, whether or not the “therapeutic” will be held up as the principle objective. Secondly, we must also make clear what type of donations we are willing to authorize from donors who are not able to give consent: only fluids and bone marrow? Or organ donation, as well? Finally, we must specify clearly the requirements for authorizing each one of these types of donations. 

Latin American countries should keep in mind that the creation of savior sibling poses dangers that must be considered when stipulating rules that will make this practice morally legitimate. Paradoxically, these dangers are related to a task that is often seen as inherently good: saving the life of a child. This contributes to the “softening” of those ethical norms that we usually have in place for organ donation from those who are legally incompetent. 

For this reason, I would like to propose an additional measure. In conjunction with requirements that have been approved in laws passed by countries that authorize the creation of SS, such as the prevention of hereditary disease in an embryo and the donation of tissues and, eventually, organs, to a sick sibling only as a last resort, I would also propose that, in order to protect the donor baby, *this donation should always be authorized by an impartial third party.* The reason for this is that in situations such as these we would be facing a typical case of justified paternalism; we cannot leave the decision in the hands of those who are most emotionally compromised, the parents. 

Stemming from this last point, our first conclusion, then, would be that any future Latin American regulation should begin by expressly defining whether compliance with the *best interest principle* should only be judged from the point of view of the parents. In contrast to this, there is also the possibility that *best interest* be judged by different regulatory authorities, such as what happens with the HFEA in the U.K, the courts in the U.S. or the National Commission on Assisted Reproduction in Spain. In each relevant case, these institutions are called upon to evaluate and rule on complex medical, social, legal, and family issues.

The imperative to intervene, found in the courts, professional associations, and ethics committees referred to above, is based, precisely, on the conviction that deciding what is in the *best interest* of a SS cannot be left only to the parents. However, there are those authors who reject these policies, arguing that the State is unjustly intervening in family autonomy and in their intimate decisions. 

Lainie Friedman Ross has criticized the *best interest standard* because it discards the “intimate nature of families” and denies the rights of parents to decide in the name of their child. The well-being of the entire family would not, necessarily, be the same as the best interest of the child, “because families can have interests that are not reducible to the interests and needs of particular members, parents must be allowed to make intrafamilial *tradeoffs* ^22^ *.* This is what she has called the “intrafamilial principle” ^23^.

The problem with this idea is that it implies accepting that parents can sacrifice one of their family members for the good of another, which in moral philosophy would be defended under the arguments of “utilitarianism” or “consequentialism.” For this moral theory, an ethical act is that which produces, collectively, the best overall result, without factoring in aspects like equity, equality, or the harm principle, among others.

A different approach, based on deontological bioethics, proposes adopting a position that respects Kant’s moral imperative of “never using people as means, but only as ends to themselves”. This philosopher reminds us that the difference that exists between things and people is directly related to the fact that things have a value relative to them that we call “price”, but people have an absolute value that we call “dignity”, and which makes them inviolable subjects that cannot be sacrificed in the name of the common good and social well-being.

The deontological moral principle, which sustains the same idea as “human rights,” forces us to admit that the actions that produce good results are not always morally justified. In order to evaluate the ethical category of an action we must focus on *how* those results were obtained. For example, although doing medical experiments on vulnerable populations can produce results that benefit the rest of society; those experiments are not ethically justifiable, because they violate human dignity.

It is for this reason that I propose a limit to the concept of “family authority”, as it is a typical case of “justified paternalism,” in which it is clear that those who must make decisions -the parents- are too emotionally compromised to be able to adequately reflect about the risks to the future child. This means putting limits to the “autonomy” of the family, since the parents, ensconced in a liminal situation, will have to take for granted that any harm caused to the SS will be justified. This does not mean that parents will not love and care for the SS (in fact, this is the norm) ^39^. Nevertheless, it does mean admitting that there exists what legal experts call a “defect of consent” (such as that which happens in the case of selling organs). That is to say, a consent that is permeated by strong emotions, and, so, not truly free nor conscious.

ii) Secondly, I propose that the Latin American countries that are still without express laws concerning organ donation on the part of children and other legally incompetent people, should create regulations that comply with, at least, these following requirements:

- that the recipient be a sibling of the donor;
- that the donation has, as its explicit goal, saving the life of the recipient;
- that the authorization, both by the legal representative as well as the institution charged with reviewing the legitimacy of the procedure, be well articulated and that it be communicated specifically in writing;
- that there exist evidence that the potential donor and the recipient will be benefitted (best interest principle);
- that the surgical risk for the donor be extremely low;
- that all other resources have been exhausted (there is no possibility of using an adult’s organs); and,
- that they minimize as much as possible the psychological and emotional risks for the child.

Finally, we should consider that some laws stipulate that in order to authorize the Pre-Implantation Genetic Diagnosis, there must be “therapeutic” ends. This would mean that the selection of an embryo would primarily be based on liberating a future child from an inherited genetic disease. From a moral point of view, this requirement does not appear to me to be entirely necessary, as the benefit that the savior sibling procedure has for the child is his/her very existence. However, this does not mean that extreme situations could not occur, for example, those of “therapeutic shackling” wherein a child could be gravely hurt by his/her parents, based on the idea that he or she was “only” created in order to be a lifelong donor for his/her sibling (these cases are known by the term *wrongful birth*). At the same time, I do not see how PGD and therapeutic ends have much to do with this, and it would not help to prevent these situations in any way.

## Conclusion

Since it is highly likely that the procedures used to create savior sibling (and the associated consequences in terms of organ donation) will continue to develop unabated, it would be a very good idea for Latin American legislatures, courts, ethics committees and healthcare operatives to agree on norms and standards that would impede excessive arbitrariness and potential abuses. In other words, if the decisions made in each case depend, in the end, on the different definitions present in the rules concerning substitute consent, the best interest principle, “acceptable” harm, family interest, etc., then an effort should be made to agree on a regulation that sets out clear guidelines and that takes advantage of comparative experiences in other countries and regions. 

Underscoring the importance of this conclusion, I would like to close with a revision of the famous Nancy Curran case, which, as we shall see, posits an important rebuttal to the Ross’ “intrafamilial principle.” This case, which was resolved by Illinois courts in 1990, involved asking for “bone-marrow testing and harvesting procedures between 3-year-old twins and their 12-year-old half-brother, who was suffering from mixed lineage leukemia” ^24^. The twins’ mother, Nancy Curran, denied consent, while the father of the twins, Tamas Bosze, authorized it. This dispute between parents ended up in the Illinois Supreme Court, which ruled in favor of Curran.

This case presented many interesting issues related to the veracity of the “intrafamilial principle”. The first issue is that the parents of possible infant organ donors may disagree about what constitutes “family interest”. The second issue concerns the relationship that should exist between two people if they are considered “members of the same family”, with the intention of invoking the intrafamilial principle. In this case, the twins were only biologically related to their half-brother, but he did not live with them and, in fact, they barely knew him. The Illinois Supreme Court argument followed in this vein, ruling that, in order for donation to be permitted, there should be a close relationship between donor and recipient ^24^.

One can agree, or not, with this court’s decision, but what is clear is that one cannot rely solely on the intrafamilial principle when deciding on the well-being of savior sibling, as this could be dangerous or, even, unnecessary. We should not forget that the case of SS is not the only one in which State intervention in the family is required. On the contrary, the majority of Western societies impose limits on what parents may do with their children. Those limits are based on the sad reality that parents may abuse or neglect their children, and, so, the State may have to intervene and take custody from them, particularly when the health and safety of the child are threatened.

For this reason, I believe that it is absolutely fundamental that countries that may develop this procedure in the future also propose regulations that recognize the necessity of involving an impartial third party in final decisions, for example, ethics committees, courts, or regulatory authorities, like the HFEA in the U.K. Final decisions with regard to organ donation on the part of children or those who are legally incompetent are very delicate and deserve being ruled on by an impartial third party. 

In the end, we should always remember the wise words of the bioethicist Jeffery Kahn: “*We know people will do anything to save their child. Now we are learning what 'anything' really means”* ^25^ *.*
