# Supplementary material for: Born to donate: proposals for “savior sibling” regulation in Latin America
Source: Colomb Med (Cali). 2018 Sep 30;49(3):228–35. doi: 10.25100/cm.v49i2.3619 (PMC6220490; doi:10.25100/cm.v49i2.3619)
Supplement: Related texts

# Research article: Niños creados para servir de donantes de órganos: propuestas para una regulación en América Latina

## Introducción

Los avances de la medicina y la investigación biomédica suelen producirse antes que la reflexión bioética sobre su pertinencia moral. Eso genera que, frente a hechos consumados, las alertas de filósofos, políticos y teóricos del derecho parezcan ya inútiles. Pocas veces podemos prepararnos para enfrentar la vorágine llamada “avance científico” y el caso de los *Saviour Sibling* (“hermano salvador”) no es la excepción. 

El término *Saviour Sibling* describe generalmente a un bebé que se crea usando la fertilización in vitro (FIV), previo al proceso de Diagnóstico Preimplantacional genético (DPG), para actuar como donante de su hermano enfermo. Como las células madre hematopoyéticas se encuentran en la médula ósea, la sangre periférica y la sangre del cordón umbilical, el hermano será utilizado, desde el momento de su nacimiento, para donar la sangre del cordón umbilical o procedimientos más invasivos tales como transfusiones repetidas de médula ósea o incluso trasplantes de órganos ^1^.

El Diagnóstico genético preimplantacional para crear niños donantes es una técnica que aún no se ha desarrollado en Latinoamérica, por lo que es pertinente -y es el objetivo de este trabajo- analizarla y considerar los peligros que implica flexibilizar las normas éticas universalmente consensuadas para la donación de órganos de incapaces. 

Aunque la creación de “saviour siblings” ha sido objeto de un extenso debate bioético, lo cierto es que un número importante de expertos y especialistas han concluido que se trata de una técnica éticamente admisible ^2^^-^^7^. Además, es una tecnología que se ha incorporado legalmente en varias legislaciones en América del Norte, Europa y Australia ^5^^-^^8^. Por ello, sin perjuicio de que la discusión bioética sobre la legitimidad moral de este procedimiento pueda y deba seguir abierta, en este artículo me concentraré en las reglas básicas que debieran definir aquellos países que se decidan a autorizar y regular la creación de “hermanos salvadores”*,* teniendo presente lo que se ha llamado *restricción de viabilidad,* que advierte sobre aquellas teorías que plantean exigencias morales poco realistas que ignoran la psicología humana y los incentivos monetarios o institucionales bajo las cuales actúan los individuo ^9^.

Considerando que se trata de un “avance científico” que llegó para quedarse y que ha sido defendido con vehemencia desde distintos frentes, tanto judiciales, bioéticos como médicos, no parece razonable insistir en su prohibición, de modo que en este trabajo no serán, más que indirectamente, analizados los siguientes argumentos en contra de la creación de *Savior Sibling*:

1. El DPG y la FIV son procedimientos éticamente cuestionables pues implican la *destrucción de embriones humanos* ^10^^).^
2. Las niñas y niños no pueden ser tratados como objetos pues ello vulnera el *principio moral de Kant* que señala “Nunca deberás usar a las personas solo como medios sino que como fines en sí mismos” ^11^.
3. El argumento de que permitir la técnica para producir *Savior Sibling* conducirá a una *pendiente resbaladiza* que terminará promoviendo el diseño de bebés ^12^^,^^13^

Luego, el objetivo del presente trabajo es defender la necesidad de que la autorización para los procedimientos que involucran la creación de los SS se garantice la intervención de un tercero imparcial capaz de velar por el bienestar del futuro niño. Para ello el artículo se divide en tres partes. Primero, un apartado que busca explicar en qué consiste el procedimiento de creación de SS, su historia y cuáles son los problemas bioéticos que se han identificado por la literatura y la práctica médica. En un segundo apartado se revisa la regulación de aquellos países que, de una u otra manera, han autorizado las técnicas de creación de SS -el DGP y la FIV. Se describen los requisitos que la legislación, tribunales y comités de ética han exigido para regular la técnica y como ellos han ido evolucionando. Finalmente, se analizan las posiciones contrarias a la tesis del trabajo y se concluye la importancia de considerar siempre la opinión de un tercero imparcial a la hora de permitir la donación de órganos o tejidos por parte de un niño o incapaz. 

## 1. Saviour Siblings: la creación 

David Benatar define las intervenciones pediátricas no terapéuticas -como la extracción de médula ósea de un bebé- como intervenciones médicas que no tienen como propósito prevenir o curar enfermedades. No buscan el beneficio del niño sobre el que se realizan sino que el de alguien más ^14^^).^ El problema moral de las intervenciones pediátricas no terapéuticas deriva del hecho de que caen fuera del límite extensamente aceptado como condición mínima irrefutable de toda injerencia médica sobre personas que no pueden consentir. Los niños, evidentemente, no pueden manifestar un consentimiento válido a temprana edad, por lo que sus representantes legales deben hacerlo cuando se trata de tratamientos que les benefician directamente, es decir, existen casos de paternalismo justificado. En cambio, las intervenciones que *no presentan un beneficio directo* sobre la niña o niño son más difíciles de justificar y ese fue el caso que se relata a continuación.

El año 1996 la hija de 4 años de Lisa y Jack Nash, Molly, fue diagnosticada con una severa enfermedad genética conocida como la anemia de Fanconi (AF). Ella afecta la producción de hematíes, leucocitos y plaquetas, facilitando el desarrollo de hemorragias, así como fallos en la médula ósea. La única cura conocida hasta hoy para la AF es el trasplante de células madres hematopoyéticas (CMH), por lo que se hacía indispensable hallar un donante con una coincidencia genética perfecta que le permitiera reemplazar su sistema inmune. 

Los Nash, motivados por la propuesta de su médico, se decidieron a emplear el innovador procedimiento de diagnóstico genético preimplantacional (DGP), que les permitiría concebir un bebé libre de la patología hereditaria que afectaba a Molly y genéticamente compatible con ella, para así poder usar su sangre del cordón umbilical para transfundir a su hermana ^15^. Fruto de este proceso, el 29 de agosto del año 2000, nacería en los Estados Unidos de Norteamérica el primer *Savior Sibling* del mundo, llamado Adam, quien con el trasplante salvaría la vida de su hermana ^16^. 

El caso de la familia Hashmi se dio en el Reino Unido, donde se demandaba autorización para la creación de un *Savior Sibling* que permitiera curar la enfermedad del niño Zain Hashmi, con un grave desorden genético en la sangre que le exigía transfusiones regulares y grandes dosis de medicamentos. Todo ello -se esperaba- podría descartarse con una transfusión de células madres del cordón umbilical de un *hermano salvador*.

Como en el caso de la familia Nash, los Hashmi estaban motivados por dos factores: concebir a un niño libre de una enfermedad genética y garantizar que ese nuevo bebé pudiese ser un donante apto para su hermano. La autoridad sanitaria del Reino Unido (The Human Fertilisation and Embryology Authority, HFEA) dio su aprobación al procedimiento destacando que se cumplía con el requisito básico para permitir el diagnóstico genético preimplantacional: el “terapéutico”, es decir, seleccionar un embrión libre de enfermedad genética heredada. En otro caso, en cambio, siguiendo ese criterio, la HFEA negó la autorización a la familia Whitaker, pues su hijo enfermo (Charlie) no tenía una enfermedad hereditaria cuya prevención pudiese justificar el Diagnóstico Preimplantacional Genético ^17^.

## 2. Saviour Siblings: la regulación

La demanda de DGP para el trasplante de células madre de niños en situaciones similares a las de Nash se disparó después de la exitosa experiencia y, por cierto, es probable que siga creciendo. Las intervenciones que se realizan son, frecuentemente, la extracción de sangre, el trasplante de médula ósea y el trasplante de órganos. Estos dos últimos casos, evidentemente, suponen un cierto riesgo y daño al bebé, sin un beneficio directo para él.

De 15 países en Europa occidental, 13 permiten DGP. Algunos, incluidos el Reino Unido y Francia, lo han hecho durante unos 20 años ^18^. Los países más recientes en permitirlo son Alemania -donde es legal desde 2011- y Suiza, que lo permite desde septiembre del año 2017 (la medida fue el resultado de un referéndum) ^19^. En América Latina se puede mencionar el caso de Brasil y el Decreto N°1321 de Diciembre del 2015 -*Regulaciones técnicas del Sistema Nacional de Trasplante*- que dispone que “el uso de células madre hematopoyéticas (médula ósea, sangre periférica o sangre del cordón umbilical) debe considerar los riesgos para el donante y los riesgos y beneficios para el receptor” ^20^.

En general, por tanto, la reglamentación de esta técnica se centra en dos aspectos relacionados con el bienestar del futuro bebé. Primero, que el DGP demuestre un objetico terapéutico y segundo, que el consentimiento sustitutivo de la voluntad del *Savior Sibling* pruebe cumplir con el criterio del “mejor interés”, propio de los casos de sustitución de consentimiento de incapaces. Es este último caso, es necesario garantizar que el beneficio para la niña o niño es mayor que el riesgo de la intervención y que no se trata de un daño excesivo.

### 2.1 El objetivo terapéutico del DGP

La regulación en el Reino Unido, España y Australia consideran necesario cumplir con los dos requisitos recién mencionados para autorizar la creación de un hermano salvador, es decir, el objetivo terapéutico y el cumplimiento del principio del mejor interés. En los Estados Unidos de Norteamérica, que no posee regulación federan sobre la materia, la autorización se centra en el segundo requisito, el del “mejor interés”, que revisaremos en el apartado siguiente ^21^.

En Australia, por su parte, aun cuando la reglamentación varía en cada Estado, la regla general es permitir el DGP sólo cuando busca, además de la compatibilidad genética con el hermano enfermo, la prevención de una enfermedad hereditaria que suponga “un riesgo real de que el futuro embrión sufra una enfermedad genética grave” ^17^. Similar era la regulación en el Reino Unido antes de la modificación que hizo la “The Human Fertilization and Embryology Authority” (HFEA) el año 2005, por la que se dispuso que “The law also permits tissue typing if the embryo will not, in addition to the histocompatibility test, be tested for a particular genetic or mitocondrial abnormality” ^22^. 

La legislación española regula el uso de la técnica en la *Ley de Técnicas de Reproducción Humana Asistida* que, en su artículo 12.2, define en qué supuestos se pueden examinar embriones para comprobar la histocompatibilidad con un tercero. A su turno, el Convenio de Oviedo establece que “De modo excepcional y en las condiciones de protección previstas por la Ley, la extracción de tejidos regenerables de una persona que no tenga capacidad para expresar su consentimiento, podrá autorizarse si se cumplen las condiciones siguientes:

- Si no se dispone de un donante compatible capaz de prestar su consentimiento;
- Si el receptor es hermano o hermana del donante;
- Si la donación es para preservar la vida del receptor;
- Si se ha dado específicamente y por escrito la autorización de los representantes y del comité de ética ^23^.^ ^

El contraste entre la normativa del Reino Unido y Australia con la normativa española plantea la interrogante relacionada con el requisito de garantizar que la técnica tenga una finalidad terapéutica. Evidentemente no se puede argüir que permitir el desarrollo de un embrión sin una enfermedad genética, le produzca algún daño al futuro niño. El razonamiento es, más bien, inverso pues, en definitiva, el niño que nace de esta técnica se ve beneficiado por la simple razón de que sin ella no existiría. Ahora bien, aun cuando se han dado casos en los que se ha reclamado indemnización por permitir el hecho mismo del nacimiento en determinadas circunstancias (los casos conocidos como *Wrongful Birth y Wrongful life)* ^24^ la técnica debiera estar autorizada en tanto se garantice que se elegirá un embrión sano que será incorporado -y tratado- como un miembro más de la familia.

### 2.2 Subrogación de consentimiento y principio del mejor interés

Las reglas sobre consentimiento informado para intervenciones no terapéuticas en adultos suelen ser estrictas. El consentimiento es un elemento ético necesario para la realización de toda intervención médica y la finalidad terapéutica es, con criterio general, el objetivo de toda actuación en el campo de la medicina. Por ello, la mayoría de los procedimientos en esta materia -exceptuando algunos casos de urgencia- requieren del consentimiento libre e informado de la propia persona ^14^. En el caso de los SS, en cambio, estamos frente a una intervención no terapéutica que se realiza sin consentimiento expreso del niño (sino que, como veremos, sustitutivo). Es por esto que uno de los requisitos fundamentales que deben cumplirse para autorizar esta técnica, es garantizar el “mejor interés” del menor. Como se ha mencionado, la autorización de la técnica puede significar, desde la extracción del cordón umbilical y el trasplante de médula hasta, eventualmente, el trasplante de órganos ^18^.

Los tribunales de justicia de los Estados Unidos han empleado, en general, dos criterios distintos para resolver la legitimidad y legalidad de los procedimientos médicos en niños o incapaces. El *estándar de juicio sustituido*, que se originó en los tribunales ingleses, se aplica a aquellos casos en los que la persona fue, alguna vez, competente, de modo que el representante decidirá de acuerdo a lo que, en su opinión, el paciente habría querido en esa situación. Alternativamente, cuando se trata de individuos que nunca han sido capaces (como es el caso de los niños pequeños) se usa *el estándar del mejor interés,* derivado de la patria potestad que el derecho reconoce a los padres. El consentimiento parental en el contexto médico implica que, una vez producido el nacimiento, los padres están facultados para consentir una intervención médica no terapéutica en nombre del recién nacido ^18^. 

En el Reino Unido se ha estimado que “los mejores intereses” del niño no se circunscriben sólo a sus intereses médicos, sino que deben incluir los intereses de toda la familia y los del hermano o hermana, es decir, el criterio de análisis debe ser global ^17^^,^^25^. Luego, para decidir sobre el cumplimiento del principio del mejor interés en los casos de donación de órganos de incapaces, los tribunales sopesan los eventuales efectos psicológicos y fisiológicos derivados de la intervención, con aquellos generados por la pérdida de un hermano ^13^^,^^21^.

El principio del mejor interés no puede obviar, por cierto, considerar los riegos asociados al procedimiento médico. En los trasplantes de células madre y órganos vivos se han reportado una variedad de riesgos psicosociales y fisiológicos para ambas partes. Los estudios de donantes infantiles de HSCT demuestran el aumento de la angustia, ansiedad y una menor autoestima de los hermanos donantes, así como niveles moderados de estrés postraumático ^17^^,^^25^. Los problemas fisiológicos para los donantes infantiles suelen ser el resultado de los medicamentos utilizados para la anestesia durante el procedimiento de trasplante y los efectos adversos propios de la naturaleza del trasplante. En el caso del trasplante de médula ósea se han observado efectos como la fatiga, dolor en el sitio de la operación, lumbalgia, dolores de cabeza, náuseas, dificultad para caminar, problemas para dormir y menos comúnmente, sangrado ^20^^,^^26^. 

En el caso de los donantes de órganos, además de la desventaja evidente de vivir con un órgano menos, se han reportado riesgos de infección, discapacidad temporal o permanente y, eventualmente, la muerte ^21^^,^^27^. Junto con ello, también hay riesgo de sufrimiento psicológico, al sentirse resentidos o deprimidos como resultado de la donación o de la falla del trasplante. Por otra parte, hay quienes tienen una reacción psicológica positiva, mostrando sentimientos de cercanía con el receptor y un sentido de contribución a la familia ^13^^,^^21^.

Las asociaciones de profesionales de la salud -como la “U.S. Live Organ Donor Consensus Group” y “The American Academy of Pediatrics” (AAP)- también han considerado la legalidad y legitimidad de la donación de órganos de menores. Aunque en principio se rechaza permitir las donaciones de órganos de niños, se han admitido excepciones si se garantiza el cumplimiento de 4 requisitos:

1. Cuando existe evidencia de que el potencial donante y el receptor serán beneficiados;
2. Cuando el riesgo quirúrgico para el donante es extremadamente bajo;
3. Cuando todos los demás recursos se han agotado (no es posible usar los órganos de un adulto) y;
4. Cuando no existe tiempo ni posibilidades efectivas de conseguir un donante de cadáver. También, a veces, se ha considerado un quinto requisito: que se minimicen los riesgos psicológicos y emocionales para el niño donante ^13^^,^^21^^).^

## 3. Donación de órganos de incapaces ¿una excepción?

En los Estados Unidos los niños son, desde hace mucho, una posible fuente de órganos sólidos. Desde el primer trasplante exitoso de riñón entre gemelos idénticos en 1954 y otros 3 en 1956, la “United Network for Organ Sharing” (UNOS) ha revelado que al menos 60 niños menores de 18 años sirvieron como donantes vivos de riñón entre los años 1987 y 2000 y otros 4 como donantes vivos de hígado desde 1989. En el paradigmático caso *Little v. Little* (576 S.W.2d 493) la Corte de Apelaciones de Texas confirmó la autorización de un trasplante de riñón de una mujer de catorce años con síndrome de Down a su hermano enfermo, invocando implícitamente el derecho de “patria potestad” de los padres y el juicio general de beneficio indirecto para el donante ^28^.

Hoy se siguen registrando una cantidad considerable de donaciones de órganos entre hermanos menores de edad en los Estado Unidos. De hecho, en California, hasta el 2006, el 12 % de los donantes de órganos tenían menos de dieciocho años ^29^. El caso de los Estados Unidos no es único registrado en Norteamérica. En Canadá, el primer caso ocurrió en 1958, donde se autorizó que una niña de 15 años donara un riñón a su hermana gemela ^30^. 

La Organización Mundial de la Salud ha dispuesto que “No deberán extraerse células, tejidos ni órganos del cuerpo de un niño vivo para fines de trasplante, excepto en las raras ocasiones *autorizadas por las legislaciones nacionales*. Deberán adoptarse medidas específicas para proteger a aquellos cuyo consentimiento deberá obtenerse, de ser posible, antes de la donación. Lo que es aplicable a los menores lo es, asimismo, a toda persona legalmente incapacitada” ^31^. En la Unión Europea, los instrumentos legales más importantes que abordan la donación de órganos y tejidos son la Directiva 2004/23/ CE y la Directiva 2010/53/ UE. Debido a que su objetivo principal es establecer un marco regulador para garantizar la calidad y seguridad de los órganos, tejidos y células, las disposiciones relevantes para la donación de menores son muy generales, dejando a los estados miembros su reglamentación específica. De este modo, la donación de órganos por menores de edad está permitida en Bélgica, Irlanda, Luxemburgo, Noruega, Suecia y Reino Unido ^32^.

En Suecia, la donación de tejidos vivos y órganos por parte de menores está regulada por la Ley de Trasplantes de 1995. La donación permitida si el niño está relacionado con el destinatario previsto y si no se ha encontrado un donante adulto competente compatible ^33^. En Inglaterra, Gales e Irlanda del Norte, no existe un límite de edad para que las personas puedan ser consideradas donantes vivos ^34^ y en Noruega solo se exige que la donación de órganos vivos se haga por menores con capacidad de consentimiento ^33^. Fuera de Europa, se han dado casos de donación de riñones o hígado de niños en países como Japón y Corea del Sur ^35^^,^^36^. 

En Latinoamérica también se han producido casos de incapaces autorizados para donar órganos, incluido Brasil ^37^. Recientemente, en Argentina, en base a una jurisprudencia de 1980 -el caso Saguir y DIB, 6-980, en el que se autorizó el trasplante de riñón de un incapaz a su hermano enfermo- recientemente, una persona con síndrome de Down (Jorge Gandur) fue declarado capaz, en el informe del “Programa Nacional de Asistencia para las Personas con Discapacidad en sus Relaciones con la Administración de Justicia” (ADAJUS), para donar un órgano (riñón) a su hermano Alfredo ^38^. 

Aunque no existen casos documentados sobre la incorporación del DGP para la producción de Hermanos Salvadores en Latinoamérica, las reglas internacionales y comparadas en materia de trasplante de órganos de incapaces servirán, sin duda, a la hora de regular la incorporación de esta técnica. 

## 4. Algunas reflexiones finales

Al principio de este trabajo señalamos que el Diagnóstico Genético Preimplantacional para crear niños donantes es una técnica que -hasta donde se tiene noticia- aún no se ha desarrollado en América Latina. El objetivo de esta investigación, por tanto, fue analizar los procedimientos destinados a la procreación de un SS, su regulación y las normas éticas que se han consensuado a propósito de la donación de órganos de incapaces. Para ello, se ha hecho una revisión de algunas legislaciones que autorizan dicha técnica y que permiten concluir algunos principios y reglas básicas susceptibles de ser recogidas por los países americanos. 

i) En primer lugar, una futura regulación habrá de tomar decisiones en relación con la técnica misma de FI y DGP y luego, respecto de la donación de órganos de incapaces. Sobre lo primero, será necesario decidir si la FI y DGP se autorizará solo con el fin de procrear un *Savior Sibling* o si, además, se requerirá que se garantice como propósito principal, el terapéutico. En segundo lugar, se deberá resolver qué tipo de donaciones por parte de incapaces van a autorizarse: sólo de fluidos y médula ósea o, también, la donación de órganos. Además, habrán de especificarse con claridad los requisitos para autorizar cada una de estas donaciones.

Al momento de acordar reglas que hagan moralmente legítima la creación de *Savior Sibling*, los países latinoamericanos debieran tener presente que esta técnica envuelve un peligro que requiere ser considerado. Ese peligro consiste, paradojalmente, en el hecho de que se trata de técnicas que buscan un objetivo loable: Salvar la vida de un niño. Y eso hace que las normas éticas que usualmente se han acordado para la donación de órganos de incapaces se ablanden. Por ello, junto con los requisitos que se han consensuado en las legislaciones de aquellos países que autorizan la creación de SS -la prevención de una enfermedad hereditaria en el embrión, la donación de tejidos y eventualmente órganos para el hermano enfermo sólo como último recurso- consideramos que la protección del bebé donante *sólo puede estar completa si se autoriza, finalmente, por un tercero imparcial.* La razón de ello es que estamos ante un caso típico de paternalismo justificado que no puede dejarse en manos de aquellos individuos -los padres- emocionalmente comprometidos.

En relación con esto último estimados que, como primera conclusión, una futura regulación en América Latina debiera comenzar por definir expresamente si la decisión respecto del cumplimiento del *principio del mejor interés* habrá de sustentarse sólo en la valoración de la situación realizada por los padres, o también -inexcusablemente- considere la evaluación derivada de las distintas autoridades regulatorias, tal y como ocurre con la HFEA en el Reino Unido, los tribunales de justicia en los Estados Unidos, o la Comisión Nacional de Reproducción Humana Asistida, en España. Estas instituciones están llamadas a intervenir para valorar, en cada caso, los aspectos médicos, sociales, jurídicos y familiares.

La intervención imperativa de los Tribunales de Justicia, Asociación de Profesionales y los Comités de Ética a las que nos hemos referido más arriba se funda, precisamente, en la convicción de que la decisión sobre lo que es “en interés” del SS no puede dejarse sólo en manos de los padres. Sin embargo, hay autores que rechazan estas políticas argumentando que el Estado está, injustificadamente, interviniendo en la autonomía de la familia y en sus decisiones íntimas. 

Lainie Friedman Ross ha criticado el *estándar del mejor interés* porque descarta “la naturaleza íntima de las familias” y niega el derecho de los padres a decidir en su nombre. El bienestar de toda la familia no sería, necesariamente, lo mismo que el mejor interés del niño, pues “because families can have interests that are not reducible to the interests and needs of particular members, parents must be allowed to make intrafamilial *tradeoffs* ^22^. Es lo que se ha llamado “principio intrafamiliar” ^23^.

El problema de esta tesis es que ella implica aceptar que los padres puedan sacrificar a uno de sus miembros en beneficio de los otros, lo que en filosofía moral defiende el llamado “utilitarismo” o “consciencialismo”. Para esta teoría moral un actuar “ético” es aquel que produce, colectivamente, el mejor resultado global, sin importar como se ponderan factores como la equidad, la igualdad, el principio del daño, entre otros. 

Un enfoque distinto, sustentado en la bioética deontológica, propone adoptar un enfoque que respete el imperativo moral de Kant que reza: “nunca uses a las personas como medio, sino que como fines en sí mismos”. El filósofo nos recordaba que la diferencia que hay entre las cosas y las personas estriba en que las cosas tienen un valor relativo al que llamamos precio, pero las personas tienen un valor absoluto al que llamamos dignidad, que las hace sujetos inviolables no susceptibles de ser sacrificados en nombre del bienestar social.

Este principio moral deontológico (que sustenta la idea misma de “derechos humanos”) nos pide admitir que no siempre las acciones que producen buenos resultados están moralmente justificadas pues para evaluar la pertinencia ética de una acción debemos centrarnos en el “cómo” se produjeron esos resultados. Así, por ejemplo, aunque la realización de experimentos médicos en poblaciones vulnerables produzca resultados que beneficien al resto de la sociedad, esos experimentos no son éticamente justificables pues violan la dignidad humana.

Es por esto que consideramos necesario imponer una limitación al principio de la patria potestad, pues este es un caso típico de “paternalismo justificado”, en el que se reconoce que quienes deben tomar decisiones -los padres- están tan emocionalmente comprometidos que no son siempre capaces de reflexionar adecuadamente sobre los riesgos para el futuro niño. Esto significa poner límites a la “autonomía” de la familia pues los padres, en una situación límite, tenderán a considerar que el daño eventual causado al SS está justificado. Ello no significa presumir que los padres no amaran y cuidarán del SS (de hecho así suele ocurrir) ^39^. Sólo se trata de admitir lo que los juristas califican como “voluntad viciada” (tal y como lo está en el caso de la venta de órganos). Es decir, una voluntad impregnada de tal carga emocional que, en verdad, no es realmente libre ni consciente.

ii) En segundo lugar, proponemos que los países americanos que aún no disponen de legislación expresa que regule las donaciones de órganos de niños u otros incapaces, incorporar una normativa que cumpla con, al menos, los siguientes requisitos:

- Que el receptor sea hermano o hermana del donante;
- que la donación tenga por fin preservar la vida del receptor;
- que la autorización, tanto del representante legal como del organismo encargado de la revisión de la legitimidad de la técnica, sea fundada y haya sido dada específicamente y por escrito;
- que exista evidencia de que el potencial donante y el receptor serán beneficiados (principio del mejor interés);
- que el riesgo quirúrgico para el donante sea extremadamente bajo;
- que todos los demás recursos se hayan agotado (es decir, que no exista la posibilidad de usar los órganos de un adulto y;
- que se minimicen al máximo los riesgos psicológicos y emocionales para el niño.

Finalmente, debemos considerar que algunas legislaciones también han requerido, para autorizar el diagnóstico genético preimplantacional, que éste tenga fines “terapéuticos”, es decir, que la selección del embrión posea como propósito principal librar al futuro niño de una enfermedad genética heredada. Adelantamos que dicho requisito no nos parece, en principio, imprescindible desde un punto de vista moral, pues el beneficio que la técnica garantiza al *Savior Sibling* es, nada menos, que su propia existencia. Ahora, esto no obsta que puedan producirse situaciones extremas -conocidas como “encadenamiento terapéutico"- en las que el niño pueda ser gravemente dañado por sus padres por el hecho de haberle concebido “solo” con el fin de ser donante de por vida de su hermano (son los casos conocidos como *wrongful bird)*. Sin embargo, no se aprecia como el DGP con fines terapéuticos podría ser útil para prevenir la ocurrencia de situaciones como esta.

## Conclusiones

Puesto que es previsible que la técnica que deriva en la creación de *Savior Sibling* (y sus consecuencias asociadas en materia de donación de órganos) seguirá desarrollándose, las legislaciones, tribunales, comités de ética y demás operadores sanitarios latinoamericanos harán bien en consensuar normas y estándares que impidan una excesiva discrecionalidad y potenciales abusos. Dicho de otro modo, si las decisiones en cada caso dependen, finalmente, de las distintas definiciones que se tengan de las reglas sobre consentimiento sustitutivo, principio del mejor interés, daño “aceptable”, interés familiar, etc. se debiera hacer un esfuerzo por acordar una normativa que proporciones directrices claras y que aproveche la experiencia comparada.

Para insistir en la importancia de nuestra conclusión puede ser útil recordar el famoso caso de Nancy Curran que, como veremos, planteó un problema importante para el “principio intrafamiliar” defendido por Ross. En este caso, resuelto por los tribunales de Illinois en 1990, se pedía “bone-marrow testing and harvesting procedures between 3-year-old twins and their 12-year-old half-brother, who was suffering from mixed lineage leukemia” ^24^. La madre de los gemelos, Nancy Curran, se negó a dar su consentimiento, mientras que el padre de los gemelos, Tamas Bosze, lo autorizó. La disputa entre los padres terminó en la Corte Suprema de Illinois que falló a favor de Curran. 

Este caso plantea muchos problemas interesantes relacionados con la verosimilitud del “principio intrafamiliar”. El primero es que los padres de los posibles donantes de órganos infantiles pueden discrepar sobre lo que constituye el “interés de la familia”. El segundo problema se refiere a la relación que debe existir entre dos personas para que se los considere “miembros de la misma familia” con el objetivo de invocar el principio intrafamiliar. En este caso, los gemelos estaban solo biológicamente relacionados con su medio hermano, pero él no vivía con ellos y apenas lo conocían. El Tribunal Supremo de Illinois argumentó, luego, es esta línea, considerando que la existencia de una relación estrecha entre el donante y el receptor debía estar presente para que la donación fuera permitida ^24^.

Se puede, por cierto, estar de acuerdo o no con la de decisión del tribunal, pero lo que parece evidente es que atenerse únicamente al principio intrafamiliar para decidir sobre lo que constituye el bienestar del SS es, en mi opinión, peligroso e innecesario. No debemos olvidar que el caso de los SS no es el único donde se requiere la intervención del Estado en la familia. La mayoría de las sociedades occidentales impone límites a lo que los padres pueden hacer con sus hijos. Esos límites se fundamentan en la realidad de que los padres pueden abusar o descuidar a sus hijos, de forma que el Estado, cuando la salud y seguridad del niño se ven amenazadas, intervine incluso retirándoles la custodia. 

En vista de lo anterior, nos parece fundamental que los países que en el futuro comiencen a desarrollar esta técnica, considere una regulación que reconozca la necesidad de implicar en la decisión final a un tercero imparcial, como es el caso de los comités de ética, las autoridades regulatorias -como la HFEA en el Reino Unido- o los tribunales de justicia. Las decisiones finales en materia de donación de órganos de niños o incapaces son tan delicadas que ameritan la participación de un tercero imparcial.

Al fin y al cabo, debemos tener presente la reflexión del bioeticista Jeffrey Kahn: “*We know people will do anything to save their child. Now we are learning what 'anything' really means*” ^25^.
